# Theoretical investigation of heterogeneous wettability in porous media using NMR

**DOI:** 10.1038/s41598-018-31803-w

**Published:** 2018-09-07

**Authors:** Jie Wang, Lizhi Xiao, Guangzhi Liao, Yan Zhang, Long Guo, Christoph H. Arns, Zhe Sun

**Affiliations:** 1 State Key Laboratory of Petroleum Resources and Prospecting, China University of Petroleum, Beijing, 102249 China; 2 Harvard SEAS-CUPB Joint Laboratory on Petroleum Science, Cambridge, MA 02138 USA

## Abstract

It is highly important to understand the heterogeneous wettability properties of porous media for enhanced oil recovery (EOR). However, wettability measurements are still challenging in directly investigating the wettability of porous media. In this paper, we propose a multidimensional nuclear magnetic resonance (NMR) method and the concept of apparent contact angles to characterize the heterogeneous wettability of porous media. The apparent contact angle, which is determined by both the wetting surface coverage and the local wettability (wetting contact angles of each homogeneous wetting regions or wetting patches), is first introduced as an indicator of the heterogeneous wettability of porous media using the NMR method. For homogeneously wetting patches, the relaxation time ratio *T*_1_/*T*_2_ is employed to probe the local wettabiity of wetting patches. The *T*_2_ − *D* is introduced to obtain the wetting surface coverage using the effective relaxivity. Numerical simulations are conducted to validate this method.

## Introduction

Wettability is defined as “the tendency of one fluid to spread on or adhere to a solid surface in the presence of other immiscible fluids”^1^. This adhesion is caused by various forces, namely, the van der Waals forces, structural forces and electrostatic force^2^, which lead to interactions between fluid molecules and solid surfaces in addition to stable fluid distributions in porous media^3^. Due to differences in their surface chemical properties, porous media exhibit two primary types of wettability: heterogeneous wettability and homogeneous wettability^4–8^. Heterogeneous wettability, which is characterized by different homogeneous wetting patches, is classified into either fractional wettability (or patterned wettability) or mixed wettability based on whether the uniform wetting patches are correlated with the pore size distribution. Kovscek provided evidence for the existence of heterogeneous wettability and introduced mixed wettability for rock samples^9^. Lebedeva *et al*. demonstrated the deposition of oil components onto mineral surfaces and the formation of wetting patterns^10^.

There are three primary types of processes through which heterogeneous wettability is formed, among which one process is caused by the different wetting properties of the original mineral compositions. The other two types are caused by the adsorption of polar compounds and the deposition of organic matter derived from crude oil onto a porous surface^2,8,11^. This matter diffuses through water before absorption or deposition onto a pore wall^8,11^; these types of matter can also come into contact with pore walls directly. When oil invades an originally water-wet reservoir, the rupture of the water films attached to the rock surface causes crude oil to come into contact with the pore surface, thereby increasing the disjoining pressure and influencing the pore shapes^2,8^. Some portions of the pore walls can become oil-wet due to the deposition of polar components while the remaining portions remain water-wet, thereby forming heterogeneous or patterned wettability^8^. In the paper, heterogeneous wettability is simplified as different regions of a solid surface with homogeneous wettability. Those different homogeneously wetting regions are called wetting patches.

The determination of wettability, which affects the capillary pressure, relative permeability, and residual oil and multiphase flow, is critical for the planning of oil production and enhanced oil recovery (EOR) endeavors^6,12^. The measurement of wettability in porous media is still challenging because of their pore structures in addition to the presence of heterogeneous wettability and multiphase fluids within their pores^6^. Numerous methods, including the contact angle method, the imbibition method, and the U.S. Bureau of Mines (USBM) or Amott method, in addition to electrical and dielectric methods and nuclear magnetic resonance (NMR), have been introduced to measure wettability^6^. Some scholars have quantitatively interpreted the wettability in fast diffusion regimes using the NMR surface relaxation method, as this approach is sensitive to the surface relaxation at the interface between a fluid and a solid surface^13–18^. Meanwhile, other scholars have quantitatively interpreted the wettability using NMR by defining the wettability index as follows^19^:

1 $${I}_{w}=\frac{surface\,wetted\,by\,water-surface\,wetted\,by\,oil}{total\,surface}$$

The wettability index can be obtained by calculating the effective relaxivity, defined as $${\rho }_{wettingfliud}$$$$\tfrac{surface\,wetted\,by\,wetting\,fluid}{total\,surface}$$, which can be obtained from the 1D *T*_1_ or *T*_2_ spectra of fluid saturated in porous media^3,20–24^. Zielinski *et al*. introduced a method to obtain the effective relaxivity by fitting the restricted diffusion line^25^. Minh *et al*. determined an approach to calculate the wettability index by fitting the restricted diffusion line on a *T*_2_ − *D* map using a Pad*é* approximation^21^. Unfortunately, the wettability index obtained using either of these methods cannot fully characterize the wettability for porous media; in addition, it cannot be employed for EOR, as it does not represent the same concept as the USBM wettability index since it ignores the local wettability of each wetting patch.

Groups led by Korb, Godefroy and Faux applied different models to interpret the *T*_1_ dispersion using fast field cycling NMR (FFC-NMR)^26–30^. Faux *et al*. applied the 3*τ* model to obtain the correlation times of the bulk fluid, surface absorbed layer and exchange time between the bulk and surface layer fluids^29,30^. Korb *et al*. proposed the concept of the probing interaction intensity between fluid molecules and a solid surface^31^. He defined the temporal ratio of the surface diffusion correlation time (*τ*_*m*_) to the exchange time (*τ*_*s*_) between a surface absorbed fluid and a bulk fluid (*τ*_*s*_/*τ*_*m*_) as an affinity index to represent the interaction intensity between fluid molecules and a solid surface^31^. The difference between Korb’s model and the 3*τ* model is that Korb’s model supposed that ${\tau }_{s}\gg {\tau }_{m}$, and for 3*τ* model, there is *τ*_*s*_ ≈ *τ*_*m*_. Korb *et al*. showed that *T*_1_/*T*_2_ correlates with the above-mentioned affinity index under a low-intensity magnetic field and subsequently applied *T*_1_/*T*_2_ to examine the local wettabilities of carbonates and shales^31–33^. The *T*_1_/*T*_2_ ratio has also been applied to probe the interactions at the interface between fluids and solid surfaces in cement^34^ and to catalysis to measure the interaction intensity between fluids and solids under low-, medium- and high-intensity magnetic fields^35^ in addition to the gas absorption on solid surfaces^36^. Valori applied the *T*_1_/*T*_2_ ratio to evaluate the downhole wettability of formations and found a good linear relationship between the *T*_1_/*T*_2_ values and the modified USBM (i.e., USBM*) index^37,38^. However, a quantitative relationship between the wettability and the *T*_1_/*T*_2_ ratio has yet to be constructed. In addition, the *T*_1_/*T*_2_ parameter cannot be employed to fully evaluate the wettability, as it does not include the surface area ratio of each wetting patch.

To fully consider the issues described above, we first introduce a new parameter to represent the overall heterogeneous wettability in porous media using NMR. The proposed parameter is used to describe the relationship between the contact angles and heterogeneous wettability. This relationship between the apparent contact angle and different wetting patches (i.e., the local wettability of each uniform wetting patch) is introduced. Based on the definition of the apparent contact angle, a multidimensional NMR method is introduced to probe the surface area ratios of the wetting patches to the total surface area (SAR-W/Ts) and their intensities. A numerical simulation is then employed to validate our model.

## Theory

### Apparent contact angles and surfaces with heterogeneous wettability

Many laboratory test methods have been introduced to describe the wettability of porous media^6^. One such approach employed contact angles on flat planes by Young’s equation to represent the wettability in porous media. Using the contact angles of water droplets on flat planes, the wettability can be classified as water-wet (0°~75°), intermediate-wet (75°~105°) or oil-wet (105°~180°)^4^.

For flat planes patterned with different wetting patches (i.e., water wetting patches and oil wetting patches), the wettability can be characterized by an apparent contact angle, which can be determined via the SAR-W/Ts and the wetting intensity of each wetting patch, the relationship between which is as follows^39^:

2 $$\cos \,{\theta }_{A}={f}_{1}\,\cos \,{\theta }_{1}+(1-{f}_{1})\,\cos \,{\theta }_{2}$$

where *f*_1_ denotes the SAR-W/T with a wetting angle of *θ*_1_, *θ*_*A*_ is the apparent contact angle of the flat surface with a heterogeneous wettability, and *θ*_1_ and *θ*_2_ are the intrinsic contact angles for wetting surface 1 and wetting surface 2, respectively.

The pore surfaces within porous media characterized by heterogeneous wettability are composed of different wetting patches. The interactions between fluids and the interaction results in a balanced fluid distribution. Here, we employ the concept of the apparent contact angle on a plane containing patterned wetting patches to represent porous media with heterogeneous wettability. Therefore, in this paper, we utilize the concept of the apparent contact angle to represent heterogeneous wettability in porous media^39^, and we employ NMR to obtain the parameters required to calculate the apparent contact angle, namely, the SAR-W/T and the local wettability of each wetting patch. These wettability indicators for porous media using NMR are different from the wettability definition in equation 1 because it is directly related to the contact angle via the SAR-W/T and the local wettability of each wetting patch. In the following sections, we consequently introduce an approach to acquire the SAR-W/T and local wettability of each wetting patch using an NMR method.

### Effective relaxivity and the SAR-W/T

Kleinberg introduced the application of the distribution of either *T*_2_ or *T*_1_ to describe the pore size distribution by assuming a uniform distribution of paramagnetic impurities on a pore surface^40^. At the pore scale, the bulk fluid and surface fluids both contribute to the relaxation time. Surface fluids constitute a thin layer with a thickness *δ* composed of absorbed fluid molecules. The total relaxation time can be expressed as follows^41,42^:

3 $$\frac{1}{{T}_{\mathrm{1,2}}}=\delta \frac{S}{V}\frac{1}{{T}_{\mathrm{1,2}S}}+(1-\delta \frac{S}{V})\frac{1}{{T}_{\mathrm{1,2}B}}$$

where subscripts 1 and 2 represent longitudinal and transverse relaxation, respectively; *S* and *V* denote the surface area and volume of the pore space; *T*_1,2*S*_ represents the intrinsic surface relaxation time; and *T*_1,2*B*_ represents the bulk relaxation time. Here, we allocate $\delta \frac{1}{{T}_{\mathrm{1,2}S}}$ to represent the relaxivity *ρ*_1,2_ ${\rho }_{1,2}=\delta \frac{1}{{T}_{1,2S}}$. In addition, $(1-\delta \frac{S}{V})\approx 1$, as *δ* is very small. Thus, equation 3 can be rewritten as

4 $$\frac{1}{{T}_{1,2}}={\rho }_{1,2}\frac{S}{V}+\frac{1}{{T}_{1,2B}}$$

Wettability, which can diminish the motion of absorbed fluid molecules^43^, is the result of various interactions between fluid molecules and a solid surface. An absorbed layer resulting from wettability with a reduced mobility is equivalent to a viscous layer. Thus, the relaxivities for surfaces with different wetting properties are different because the influences of the local wettability on the motions of fluid molecules are different. This feature is therefore used to discern different wetting patches.

If a strongly water-wet rock system is uniformly wetted, a water film will form on the rock surface. This film will prevent oil from interacting with the solid surface^2^. Thus, oil will process bulk relaxation, and water will process surface relaxation and vice versa^16,40^. When the water or oil saturation changes, water will still preferentially come into contact with the pore surface while oil enters the pores.

This can be extended to a porous media system with heterogeneous wettability. When porous media characterized by heterogeneous wettability are partially saturated with both water and oil, the water will preferentially come into contact with water-wet patches, while oil will preferentially come into contact with oil-wet patches. The total surface relaxation time in partially saturated porous media with heterogeneous wettability is

5 $$\frac{1}{{T}_{2S}}={\rho }_{w}\frac{{S}_{w}}{{V}_{w}}+{\rho }_{o}\frac{{S}_{o}}{{V}_{o}}={\rho }_{w}\frac{{S}_{w}}{S}\frac{1}{({V}_{w}/V)}(\frac{S}{V})+{\rho }_{o}\frac{{S}_{o}}{S}\frac{1}{({V}_{o}/V)}(\frac{S}{V})$$

where the subscripts *w* and *o* denote water and oil, respectively; *S*_*i*_ represents the surface area between a fluid *i* and a solid surface; and *V*_*i*_ is the volume of the phase *i*. The saturation of the fluid *i* is ${s}_{i}=\frac{{V}_{i}}{V}$. The SAR-W/T for the fluid *i* and the solid surface is defined as

6 $${I}_{i}=\frac{{S}_{i}}{S}$$

Thus, equation 5 can be rewritten as follows:

7 $$\frac{1}{{T}_{2S}}={\rho }_{w}\frac{{I}_{w}}{{s}_{w}}(\frac{S}{V})+{\rho }_{o}\frac{{I}_{o}}{{s}_{o}}(\frac{S}{V})={\rho }_{effw}\frac{1}{{s}_{w}}(\frac{S}{V})+{\rho }_{effo}\frac{1}{{s}_{o}}(\frac{S}{V})$$

where *ρ*_*effi*_ = *ρ*_*i*_*I*_*i*_, which we define as the effective relaxivity of the phase *i*. The effective relaxivity is clearly linearly proportional to the SAR-W/T. Thus, we can acquire the SAR-W/T, which is required for the calculation in equation 2, from the effective relaxivity. Evidently, *D* − *T*_2_ is sensitive to the effective relaxivity, and the fluids can be readily distinguished from the *D* − *T*_2_ map^25^; therefore, we introduce an approach employing the *D* − *T*_2_ map to obtain the effective relaxivity in the following section.

### Determination of the effective relaxivity from the *D* − *T*_2_ map

We introduced the relationship between *ρ*_*eff*_ and the SAR-W/T above. In this section, we aim to obtain *ρ*_*eff*_ from the *D* − *T*_2_ map, from which different fluids can be easily differentiated. The *D* − *T*_2_ map is additionally convenient because it provides a comparatively precise method for calculating *ρ*_*eff*_^25,44^.

The motion of fluid molecules within a bulk fluid follows Brownian motion. The diffusion coefficient evolving with time is calculated as

8 $$D(t)=\frac{\langle {[{x}_{t}-{x}_{0}]}^{2}\rangle }{2dt}$$

where 〈[*x*_*t*_ − *x*_0_]^2^〉 is the mean-square displacement (*x*) of a fluid molecule during the time *t* from its initial position at a time of zero, and *d* is the number of dimensions. The diffusion of fluids in porous media is different from that in a bulk fluid because the presence of a solid wall limits Brownian motion. If the diffusion time is sufficiently long such that the diffusion distance of a molecule is far larger than the pore size, the diffusion coefficient in a porous medium tends to be a constant:

9 $${D}_{\infty }={D}_{0}/\tau $$

where *D*_0_ is the intrinsic self-diffusion coefficient and *τ* is the tortuosity of a porous medium, defined as the ratio of geometric flow paths through the medium to the straight-line length of the medium. For a short diffusion time in which the diffusion length is much smaller than the scale of the pore size, only molecules located within $\sqrt{{D}_{0}t}$ of the boundary sense the boundary. These molecules represent a proportion $\sqrt{{D}_{0}t}S/V$. The apparent diffusion coefficient for short diffusion times in porous media has been given^45^:

10 $$\frac{D(t)}{{D}_{0}}=1-\frac{4}{9\sqrt{\pi }}\frac{S}{V}\sqrt{{D}_{0}t}$$

For a partially saturated porous medium, the parameters *D*_0_, *S* and *V* are unique for each fluid considered. To relate short diffusion times with long diffusion times, a Pad*é* approximation is applied and replace *S*/*V* with 1/*ρT*_2_. Here, we neglect bulk relaxation because the surface relaxation time is much shorter than the bulk relaxation time in porous media^18,44^. Thus, *D*(*t*) is a function of *T*_2_. At each *T*_2_ value, we have^44^

11 $$D({T}_{2})={D}_{0}\,(1-\beta \frac{a{L}_{D}+{({L}_{D}/{L}_{M})}^{2}}{a{L}_{D}+{({L}_{D}/{L}_{M})}^{2}+\gamma })$$

where *β* = 1 − *D*_∞_/*D*_0_, $a=\frac{4}{9\sqrt{\pi }}\frac{1}{{T}_{2}\rho }$, ${L}_{D}=\sqrt{{D}_{0}t}$ (where *t* is the diffusion time), and *L*_*M*_ is the heterogeneity length scale of the medium. Since *L*_*M*_ will be much larger than *L*_*D*_, (*L*_*D*_/*L*_*M*_)^2^ is negligible. Equation 11 is for uniformly distributed surface relaxivity. With a heterogeneously distributed surface relaxivity, the apparent diffusion coefficient is a function of *T*_2_ and the surface relaxivity *ρ*_*eff*_ (*ρS*/*V*), that is, *D*(*T*_2_, *ρ*_*eff*_, *t*).

Thus, the measured *D* and *T*_2_ distribution should follow the Pad*é* approximation equation 11. By fitting *D*_*i*_ and *T*_2*i*_ on the *D* − *T*_2_ map with the Pad*é* approximation for each fluid at each *T*_2_ value (*T*_2*i*_), the effective relaxivity can be obtained:

12 $${\rm{\min }}\,\sum _{i}\,{w}_{i}{(D({T}_{2i},{\rho }_{eff})-{D}_{i})}^{2}$$

where the subscript *i* represents the *i*^*th*^ component of *T*_2_ and *w*_*i*_ is a weighting function. Here, we use the weight of each *T*_2_ component to represent *w*_*i*_.

Two parameters, namely, *s*_*w*_ and *β*, must be calculated. In the case of mixed saturation with two phases, we apply a log-mean diffusion approach to separate the fluid signals and obtain the water saturation^46^:

13 $${s}_{w}({T}_{2})=\frac{\mathrm{ln}({D}_{LM}({T}_{2})/{D}_{0}({T}_{2}))}{\mathrm{ln}({D}_{w}({T}_{2})/{D}_{0}({T}_{2}))}$$

where *D*_*LM*_(*T*_2_) is the logarithmic mean of the diffusion coefficient at each value of *T*_2_ and *D*_*w*_(*T*_2_) and *D*_0_(*T*_2_) denote the water diffusion coefficient and oil diffusion coefficient, respectively, at each *T*_2_ value. *D*_∞_/*D*_0_ is obtained by PFG-CPMG^45^.

### *T*_1_/*T*_2_ and local wettability

**Figure 1:**
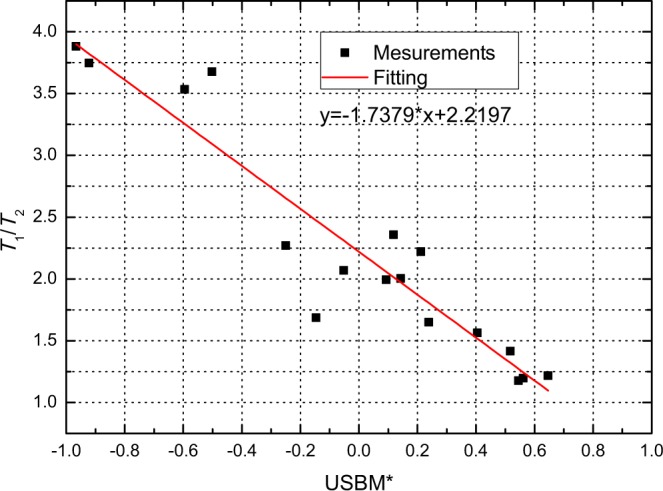
Correlation between averaged *T*_1_/*T*_2_ values of the oil phase and water phase and the measured USBM* wettability index (data from^38^).

The value of the ratio *T*_1_/*T*_2_ is an indicator of the intensity of the interaction between the fluid molecules and the solid surface (i.e., the wettability). Valori employed the ratio *T*_1_/*T*_2_ with a measurement frequency of 2 MHz to evaluate formation wettabilities and correlated the resulting *T*_1_/*T*_2_ values with with USBM* index (i.e., the modified USBM index, detailed in^38^) as shown in Fig. 1. This empirical relationship is also applied for the water phase^34,38^. Here, we use the cosine values of the contact angles to represent the local wettability. Thus, we can apply the *T*_1_/*T*_2_ values to denote the local wettability of wetting patches.

## Simulation Methods

The Shan-Chen Lattice Boltzmann (SC LBM) model can be utilized to simulate fluid distributions with different wettability properties in porous media^47,48^. In this section, we first apply the SC LBM model to validate equation 2 by measuring the apparent contact angles of simulated droplets with varying SAR-W/Ts and local wettability of wetting patches on flat planes. Then, we apply the concept of apparent contact angles to porous media.

To validate the multidimensional NMR method used to obtain the parameters required to calculate the apparent contact angle, we perform the following simulation experiment. First, the porous media models employed in this simulation are constructed using the sphere packing method. Porous media models with different wettability properties are obtained by varying the ratios of different wetting spheres and the wetting properties of those spheres. Under different wetting conditions, the SC LBM is employed to simulate the fluid distributions, which are the results of interactions between the fluids and wetting patches. Based on those fluid distributions, the random walk method is applied to stimulate the oil and water response inside porous media with heterogeneous wetting properties. A hybrid inversion-recovery pulse sequence, namely, a pulsed field gradient plus Carr-Purcell-Meiboom-Gill (PFG-CPMG) pulse sequence, is employed to obtain the *T*_1_ − *T*_2_ − *D* maps, from which *D* − *T*_2_ and *T*_1_ − *T*_2_ maps can be obtained.

### Porous media with heterogeneous wettability

Porous media characterized by a heterogeneous wettability are simplified into models constructed by randomly packing spheres with different radii in a 200 × 200 × 224 um box. The radii of these balls follow a normal distribution with an average radius of 10 um and a standard deviation of 0.1, which limits the NMR measurements to a fast relaxation regime. The spheres are established as being either oil-wet or water-wet with different wettability. Consequently, by varying the ratios of different wetting spheres with different wettability, porous media with heterogeneous wettability can be established. The center part of the model is extracted as the substructure and digitized as a 200 × 200 × 200 cube with a resolution of 0.5 um per pixel. The SC LBM is applied to the interior of these substructures with heterogeneous wettability to simulate the oil and water distributions.

### The SC LBM

The SC LBM is applied to simulate the multicomponent fluid distributions in porous media based on the multicomponent interactions as well as the interactions between liquid molecules and solid surfaces. The detailed mathematics of this scheme can be found in *Shan and Chen*, *1993*. For each fluid component, the distribution and evolution equation can be described as follows:

14 $${f}_{i}^{\sigma }({\bf{x}}+{{\bf{e}}}_{i}{\rm{\Delta }}t,t+{\rm{\Delta }}t)={f}_{i}^{\sigma }({\bf{x}},t)-\frac{{\rm{\Delta }}t}{{\tau }_{\sigma }}[{f}_{i}^{\sigma }({\bf{x}},t)-{f}_{i}^{\sigma eq}({\bf{x}},t)]$$

where ${{f}_{i}}^{\sigma }({\bf{x}},t)$ is the density distribution function of the fluid *σ* in the *i*^*th*^ velocity direction at a position **x** and time *t* and *τ*_*σ*_ is the relaxation time of the fluid *σ*. The relationship between *τ*_*σ*_ and the kinematic viscosity is represented by ${\nu }_{\sigma }={c}_{s}^{2}({\tau }_{\sigma }-0.5{\rm{\Delta }}t)$ and ${c}_{s}=c/\sqrt{3}$. *c* = Δ*x*/Δ*t*, where Δ*t* denotes the time step and Δ*x* denotes the lattice spacing. The equilibrium distribution function ${{f}_{i}}^{\sigma ,eq}({\bf{x}},t)$ is written as follows:

15 $${f}_{i}^{\sigma ,eq}({\bf{x}},t)={w}_{i}{\psi }_{\sigma }[1+\frac{{{\bf{e}}}_{i}\cdot {{\bf{u}}}_{\sigma }^{eq}}{{c}_{s}^{2}}+\frac{{({{\bf{e}}}_{i}\cdot {{\bf{u}}}_{\sigma }^{eq})}^{2}}{2{c}_{s}^{4}}-\frac{{{\bf{u}}}_{\sigma }^{eq}}{2{c}_{s}^{2}}]$$

For the D3Q19 model, the discrete velocities **e**_*i*_ are given by the following matrix:

16 $${\bf{e}}=c\,[\begin{array}{ccccccccccccccccccc}0 & 1 & -\,1 & 0 & 0 & 0 & 0 & 1 & 1 & -\,1 & -\,1 & 1 & -\,1 & 1 & -\,1 & 0 & 0 & 0 & 0\\ 0 & 0 & 0 & 1 & -\,1 & 0 & 0 & 1 & -\,1 & 1 & -\,1 & 0 & 0 & 0 & 0 & 1 & 1 & -\,1 & -\,1\\ 0 & 0 & 0 & 0 & 0 & 1 & -\,1 & 0 & 0 & 0 & 0 & 1 & 1 & -\,1 & -\,1 & 1 & -\,1 & 1 & -\,1\end{array}]$$

where *w*_*i*_ = 1/3 (*i* = 0), *w*_*i*_ = 1/18 (*i* = 1, 2, …, 6), and *w*_*a*_ = 1/36 (*i* = 7, 8, …, 18). For the D2Q9 model, the discrete velocities **e**_*i*_ are given as

17 $${\bf{e}}=c\,[\begin{array}{ccccccccc}0 & 1 & 0 & -\,1 & 0 & 1 & -\,1 & -\,1 & 1\\ 0 & 0 & 1 & 0 & -\,1 & 1 & 1 & -\,1 & -\,1\end{array}]$$

where *w*_*i*_ = 4/9 (*i* = 0), *w*_*i*_ = 1/9 (*i* = 1, 2, 3, 4), and *w*_*a*_ = 1/36 (*i* = 5, 6, 7, 8). *ψ*_*σ*_ is equal to the density of the fluid *σ*, and it is calculated according to ${\psi }_{\sigma }={\sum }_{i}\,{{f}_{i}}^{\sigma }$. **u**^*σ*,*eq*^ is given by

18 $${{\bf{u}}}^{\sigma ,eq}={\bf{u}}^{\prime} +\frac{{\tau }_{\sigma }{{\bf{F}}}_{\sigma }}{{\psi }_{\sigma }}$$

where **u**′ is the common velocity of a multicomponent fluid, and it is given by

19 $${\bf{u}}^{\prime} =\frac{{\sum }_{\sigma }\,({\sum }_{i}\,{f}_{i}^{\sigma }{{\bf{e}}}_{i})}{{\sum }_{\sigma }\,\frac{{\psi }_{\sigma }}{{\tau }_{\sigma }}}$$

In equation 18, **F**_*σ*_ represents the combination of two interactions: fluid-fluid interactions **F**_*σ*,*c*_ and the interactions between fluids and solid walls **F**_*ads*,*σ*_. In a binary mixture, the interactive force acting on the *σ*^*th*^ component is defined as

20 $${{\bf{F}}}_{\sigma ,c}(x,t)=-\,{G}_{c}{\psi }_{\sigma }({\bf{x}},t)\,\sum _{i}\,{w}_{i}{\psi }_{c}({\bf{x}}+{{\bf{e}}}_{i}{\rm{\Delta }}t){{\bf{e}}}_{i}$$

where *c* and *σ* represent different fluid components and *G*_*c*_ is a constant value adjusting the forces between different fluid components. *G*_*c*_ controls the interfacial tension between fluids.

Wettability is considered as the result of the interactions between liquids and the walls of pore spaces. The force between a liquid and a solid surface is given by **F**_*σ*,*ads*_ as follows:

21 $${{\bf{F}}}_{\sigma ,ads}(x,t)=-\,{G}_{ads}{\psi }_{\sigma }(x,t)\,\sum _{i}\,{w}_{i}s({\bf{x}}+{{\bf{e}}}_{i}{\rm{\Delta }}t){{\bf{e}}}_{i}$$

where *G*_*ads*_ is a constant value controlling the adhesion force between the walls and fluids and *s*(**x** + **e**_*i*_Δ*t*) is equal to 0 or 1, being based upon whether it represents a pore space or a solid. The wettability intensity (i.e., wetting angle) can be obtained by adjusting the values of *G*_*ads*_ and *s*(**x** + **e**_*i*_Δ*t*). In this section, we assign different values of *G*_*ads*_ to different components of porous media characterized by heterogeneous wettability. Young’s equation, which is employed to compute the contact angles corresponding to the adhesion parameters between fluids and solid surfaces, is written as follows^48^:

22 $$\cos \,\theta =\frac{{G}_{ads,\sigma }-{G}_{ads,c}}{{G}_{c}\frac{{\psi }_{\sigma }-{\psi }_{c}}{2}}$$

To validate equation 2, we assign different SAR-W/Ts of oil-wet patches and water-wet patches to flat planes. Accordingly, by varying the candidate SAR-W/Ts and wetting intensities, the apparent contact angles of droplets on flat planes with heterogeneous wettability can be obtained. The contact angle on flat plan can be calculated with the length of base (*L*) and height (*H*) of the droplet. The radius of the droplet is $R=\frac{4{H}^{2}+{L}^{2}}{8H}$. Thus *tanθ* = *L*/2/(*R* − *H*).

### NMR response simulation

The fluid distributions in porous media with heterogeneous wettability are simulated using the SC LBM. These fluid distributions illustrate which fluid comes into contact with a solid surface during surface relaxation. Fluids with molecular spins exhibit Brownian motion, which can be simulated using a random walk method^18^. Because this approach can be used to imitate the Brownian motions of molecules, the NMR responses of fluids in porous media can be simulated using the random-walk method^49^. Initially, a large number of water and oil walkers are randomly placed within a water and oil distribution regime. These water and oil walkers diffuse with different diffusion coefficients. For each step, the direction of motion is randomly chosen as follows^50^:

23 $$\begin{array}{rcl}x^{\prime}  & = & x+{\rm{\Delta }}r\,\sin \,\theta \,\cos \,\phi \\ y^{\prime}  & = & y+{\rm{\Delta }}r\,\sin \,\theta \,\sin \,\phi \\ z^{\prime}  & = & x+{\rm{\Delta }}r\,\cos \,\theta \end{array}$$

where *x*, *y*, and *z* are the initial coordinates of a walker, and *x*′, *y*′, and *z*′ are the new coordinates after a step of length Δ*r*. The moving direction is assigned according to randomly chosen values of *θ* and *φ*. Each step requires a time Δ*t* = Δ*r*^2^/6*D*. The motions of the walkers are restricted to within the porous media; if the walkers exceed the boundaries of the sample, the parameters *θ* and *φ* are reproduced until the walkers are inside the sample. If a magnetic field gradient is applied along the *x* axis, each walker bearing a molecular spin is subjected to dephasing at each walking step^50^:

24 $$\varphi (t+{\rm{\Delta }}t)=\varphi (t)+\gamma {G}_{ext}\frac{x(t+{\rm{\Delta }}t)+x(t)-2x\mathrm{(0)}}{2}{\rm{\Delta }}t$$

where *G*_*ext*_ is the applied magnetic field gradient along the *x* axis, *x*(*t*) represents the *x* coordinate of a walker at the time *t*, and *γ* is the gyromagnetic ratio.

When walkers bearing a molecular spin encounter a solid surface, they interact with paramagnetic ions adhered onto the solid surface. This interaction causes the walkers to lose their magnetization information with an infinite possibility^49,51,52^:

25 $$p=\frac{2}{3}\frac{\rho {\rm{\Delta }}r}{D}\times 0.96$$

Details can be found in reference *Toumelin et al*.^49^.

As both *T*_1_ and *T*_2_ are correlated with the specific surface area (equation 3), the relaxation effect can be ascribed to the surface relaxivity *ρ*_1,2_^53^. For the NMR response simulation, we assign different values of the ratio *ρ*_1_/*ρ*_2_ to obtain the desired value of *T*_1_/*T*_2_. If a porous medium is saturated with two types of fluids, the parameters (e.g., *ρ* and *D*) of those fluids found in equations 24 and 25 are assigned the intrinsic value for each fluid. If walkers bearing molecular spins encounter the interface between oil and water, the walkers step back without relaxation^49^. This simulation is implemented with a working frequency of 2 MHz. To obtain and encode the information regarding *T*_1_, *T*_2_ and *D*, we apply the modified inversion-recovery IR-PFG-CPMG pulse sequence. The acquired data can be expressed as

26 $$b(t,{\rm{\Delta }},{\tau }_{1})=\int \,\int \,\int \,f(D,{T}_{1},{T}_{2}){k}_{1}(t,{T}_{2}){k}_{2}(t,{T}_{1}){k}_{3}(t,{\rm{\Delta }},D)dDd{T}_{1}d{T}_{2}$$

where *k*_1_(*t*, *T*_2_) = exp(−*t*/*T*_2_), *k*_2_(*t*, *T*_1_) = 1 − exp(−*t*/*T*_1_) and ${k}_{3}(t,{\rm{\Delta }},D)=-\,\frac{2}{3}{\gamma }^{2}{\delta }^{2}{g}^{2}D({\rm{\Delta }}-\frac{1}{3}\delta )$. *τ*_1_ is used to encode *T*_1_, Δ and *δ* are the encoding time and duration of the pulse gradient, both of which being used for encoding the diffusion *D*, and *τ*_2_ is the echo spacing in the CPMG pulse.

## Results

### Surface heterogeneity and apparent contact angles on flat plane

In this section, we aim to validate the relationship between the apparent contact angles and different wetting patches patterned on flat planes, including the association between the SAR-W/Ts and local wettability of those patches, as previously introduced.

The flat planes with heterogeneous wettability are composed of two types of wetting patches: water-wet patches and oil-wet patches. We divide the following work into two parts. For the first part, by fixing the local wettability of each wet patch and varying the SAR-W/Ts of the water-wet/oil-wet patches, we examine the relationship between the apparent contact angles and SAR-W/Ts of those water-wet patches. For the second part, by varying the local wettability of each patch, we study the relationship between the apparent contact angles on flat planes and the intrinsic contact angle of each wetting patch (local wettability).

**Figure 2:**
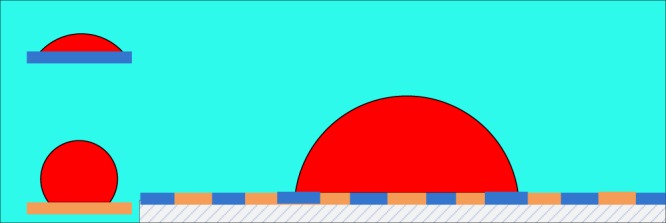
The illustration of simulation region plane with heterogeneous wettability and initially water distributed. The insertions represent water and oil wetting conditions.

For both parts of the work conducted hereafter, the simulation domain is a 100 × 100 (pixel) square. Only one of four sides is a boundary serving as a solid wall, possessing evenly distributed oil-wet patches (1 pixel) and water-wet patches (1 pixel), as shown in Fig. 2; the other boundaries are open. A half circle immersed in this 100 × 100 square represents one-half of a water droplet immersing in oil and attached to the solid surface. The radius of the half droplet is 25 pixels. The initial density of water within the halved droplet is 2.0, and that on the outside of the droplet is 0.05, which represents the dissolved density. In contrast, the density of oil within the droplet is 0.05, and that on the outside is 2.0, which satisfies the parameter selection protocol. For both fluids, *τ* = 1. We apply a bounce-back condition to the solid boundary to address the interactions with solid walls; for the open boundaries, we apply periodic boundary conditions^48,54^.

**Figure 3:**
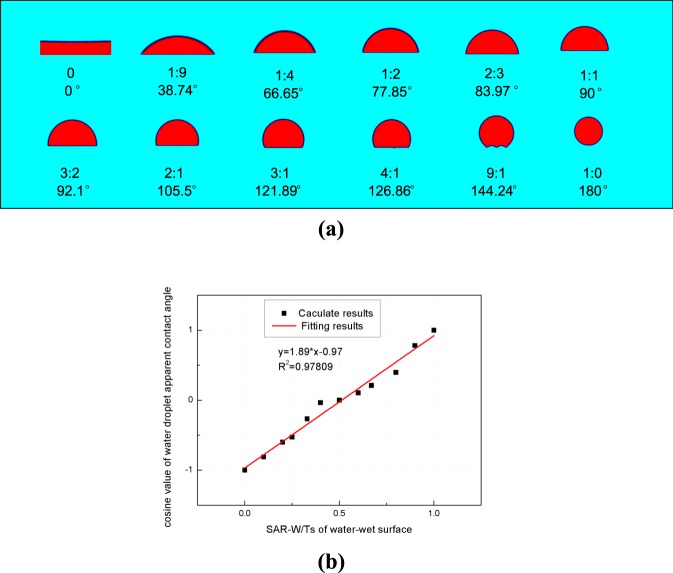
SC LBM simulation results of water droplets on flat planes with different SAR-W/Ts of the water-wet patches (**a**) and the fitting results between the apparent contact angles and SAR-W/Ts of the water-wet patches (**b**). The apparent contact angles of the simulated droplets on flat planes are calculated using the geometric relationship. The first and fourth rows of (**a**) constitute droplets on flat planes simulated using the SC LBM, the second and fifth rows of (**a**) constitute the surface area ratios of oil-wet to water-wet patches, and the third and sixth rows of (**a**) constitute the apparent contact angles calculated from the first and fifth rows of (**a**).

To simplify the first part of this work (i.e., to examine the relationship between the apparent contact angles and SAR-W/Ts), we assume that two types of wetting patches are evenly distributed along the planes (the contact angle is 0 rad). The surface area ratios of the oil-wet to water-wet patches are 0, 1:9, 1:4, 1:2, 2:3, 1:1, 3:2, 2:1, 3:1, 4:1, 9:1, and 1:0. The SC LBM is employed to simulate the water and oil distributions on the flat planes characterized by heterogeneous wettability. The coherence parameter *G*_*c*_ between the water and oil is −1.8. The parameter controlling the adhesion force between the water and water-wet surface is *G*_*adsw*_ = −1.8, and that between the oil and oil-wet surface is *G*_*adso*_ = −1.8. The calculation is repeated 20000 times, after which the density distribution variance is less then 10^−3^. The apparent contact angles are calculated using the geometric relationship. The simulation results and fitting results between the apparent contact angles and SAR-W/Ts are shown in Fig. 3.

**Figure 4:**
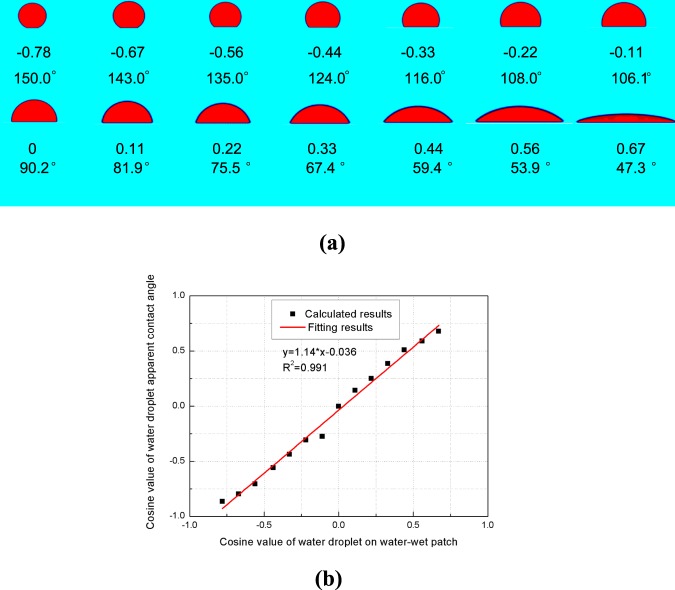
SC LBM simulation results of water droplets on flat planes with different intrinsic contact angles of water-wet patches (**a**) and the fitting results between the apparent contact angles and intrinsic contact angles of water-wet patches (**b**). The apparent contact angles of the simulated droplets on flat planes are calculated using the geometric relationship. The first and forth rows of (**a**) are droplets on flat planes simulated using the SC LBM, the second and fifth rows of (**a**) are the intrinsic contact angles of water-wet patches, and the third and sixth rows of (**a**) are the apparent contact angles calculated from the first and fifth rows of (**a**).

For the second part of this work, we vary the intrinsic contact angles of the wetting patch local wettability to obtain and examine the relationship between the apparent contact angles and local wettability of each wetting patch. For simplicity, the flat planes are set as either oil-wet or water-wet. The initial conditions and parameters for this simulation are identical to those in the first part of the simulation except that *G*_*adsw*_ = −1.8. The intrinsic contact angles of water droplets on the water-wet patches for the forward model are calculated using equation 22, which returns values of 30°, 37°, 45°, 56°, 64°, 72°, 83.9°, 90.2°, 98.1°, 104.5°, 112.6°, 120.6°, 126.1°, and 132.7°. The simulation and fitting results between the intrinsic contact angles of water-wet patches and the apparent contact angles calculated for flat planes are shown in Fig. 4.

### Simulation of the fluid distributions and NMR responses for porous media

In this section, we apply the results of section 3.1 and the concept of apparent contact angles for porous media characterized by heterogeneous wettability. To measure the apparent contact angles of porous media, a multidimensional NMR method is employed to obtain the parameters required for equation 2. Porous media models with different wettability properties are obtained by varying the ratios of different wetting spheres and the wetting properties of those spheres. The SC LBM is applied under different wetting conditions to simulate the fluid distributions in porous media. Based on those fluid distributions, a hybrid inversion-recovery PFG-CPMG pulse sequence is applied to the simulate NMR responses, and we obtain the *D* − *T*_2_ and *T*_1_ − *T*_2_ maps.

The porous media with heterogeneous wettability employed in this simulation were constructed previously in the porous media with heterogeneous wettability section. The structure is a 200 × 200 × 200 cube with a resolution of 0.5 um per pixel. To study the effects of different local wettability and different SAR-W/Ts on the fluid distributions, we assign different wetting properties to the spheres. Correspondingly, this work is also divided into two parts, namely, investigations into the effects of SAR-W/T and the local wettability of each wetting patch on the fluid distributions. Based on those fluid distributions, the NMR responses are simulated accordingly, and the parameters required to calculate the apparent contact angle are then obtained from the NMR responses.

**Figure 5:**
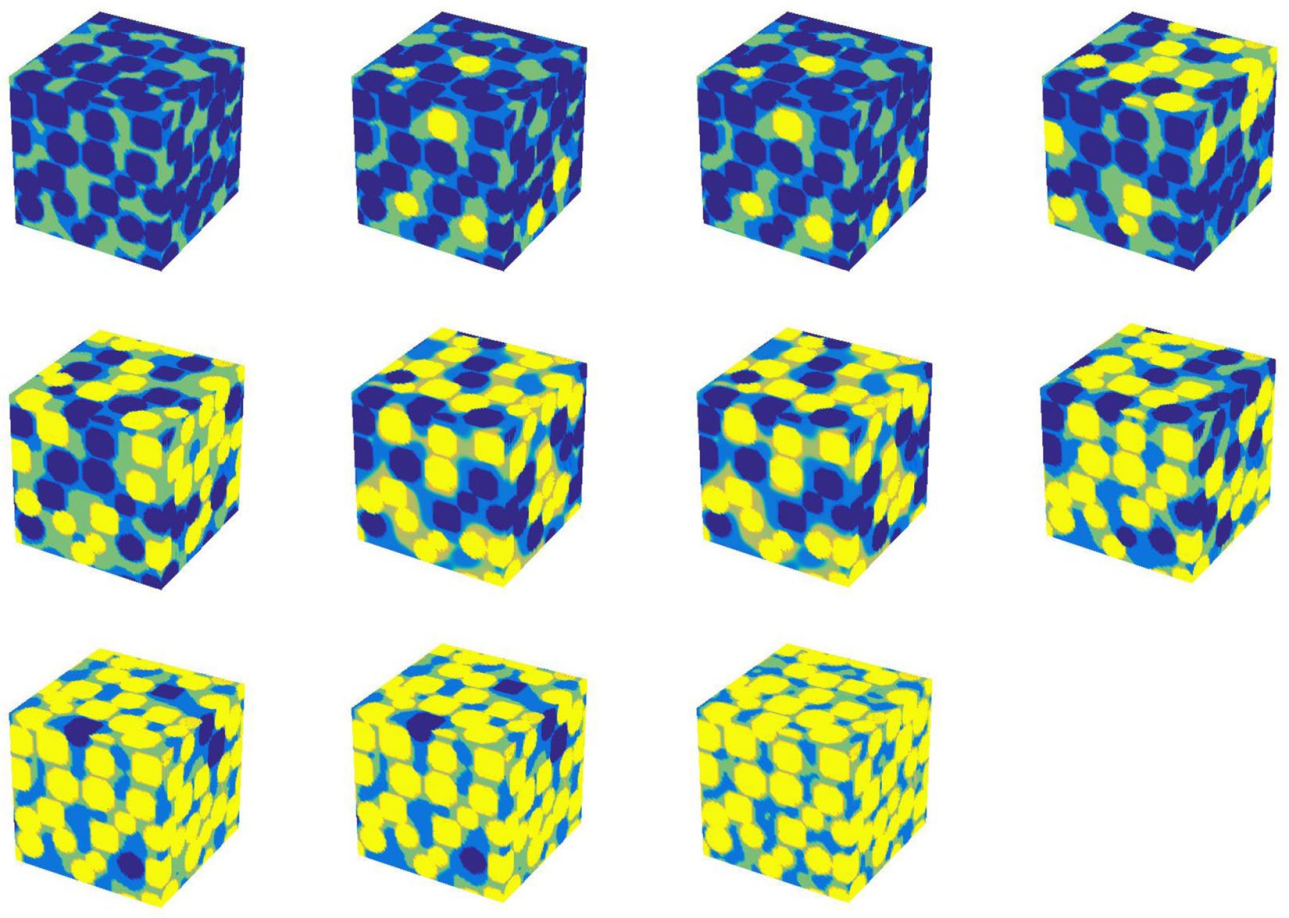
Oil and water distributions in porous media with heterogeneous wettability. The SAR-W/Ts of the water-wet surface are (from left to right and from top to bottom) 0.0, 0.09, 0.18, 0.28, 0.40, 0.47, 0.59, 0.70, 0.79, 0.89, and 1.0. The blue spheres are water-wet, and the yellow spheres are oil-wet. The light blue and light yellow parts represent the water and oil distributions, respectively.

For the first part of this section, the spheres are classified as either strongly water-wet or strongly oil-wet (the contract angle is 0 rad). The SAR-W/Ts of the strongly water-wet surfaces are 0.0, 0.09, 0.18, 0.28, 0.40, 0.47, 0.59, 0.70, 0.79, 0.89, and 1.0. The SC LBM is employed to simulate the water and oil distributions. Initially, both the oil and the water are randomly distributed throughout the pore space. The initial densities of the oil (*ψ*_*o*_) and water (*ψ*_*w*_) are both 2, and the water saturation is 50%. We set *τ* equal to 1 for both oil and water since the system does not experience flow, as was discussed in *Sukop et al*.^54^; thus, the viscosity is irrelevant. Similarly, as we are focused only on the fluid distributions, the absolute and relative densities are of no concern. The parameter *G*_*c*_, which controls the cohesion force between water and oil, is set to 1.8. The parameter controlling the adhesion force for the water-wet patches is *G*_*adsw*_ = −1.8, and that for the oil-wet patches is *G*_*adso*_ = −1.8. The calculation is repeated 20000 times, after which the density distribution variation is less than 10^−3^. The parameters employed for the fluid distribution simulation are listed in the top two rows of Table 1. The simulation results are shown in Fig. 5, which demonstrates that oil (light yellow parts) mainly comes into contact with oil-wet spheres (yellow spheres). In contrast, water (light blue color) mainly comes into contact with water-wet spheres (blue spheres).

**Table 1:** Parameters used for the fluid distribution simulation and NMR response simulation.

*ψ*_*w*_	*ψ*_*o*_	*s*_*w*_	*τ*_*w*_	*τ*_*o*_	*G*_*c*_	*G_*adsw*_	*G_*adso*_
2.0	2.0	0.5	1.0	1.0	−1.8	−1.8	−1.8
***D***_***o***_ **(um** ^**2**^ **/ms)**	***D***_***w***_ **(um** ^**2**^ **/ms)**	***T***_***2bo***_ **(ms)**	***T***_***2bw***_ **(ms)**	***ρ***_***o***_ **(um/s)**	***ρ***_***w***_ **(um/s)**	***s***_***w***_ **(%)**	
0.51	2.1	1000	3000	30	10	50	

*G_*adsw*_ and *G*_*adso*_ are the parameters for water-wet and oil-wet patches, respectively.

**Figure 6:**
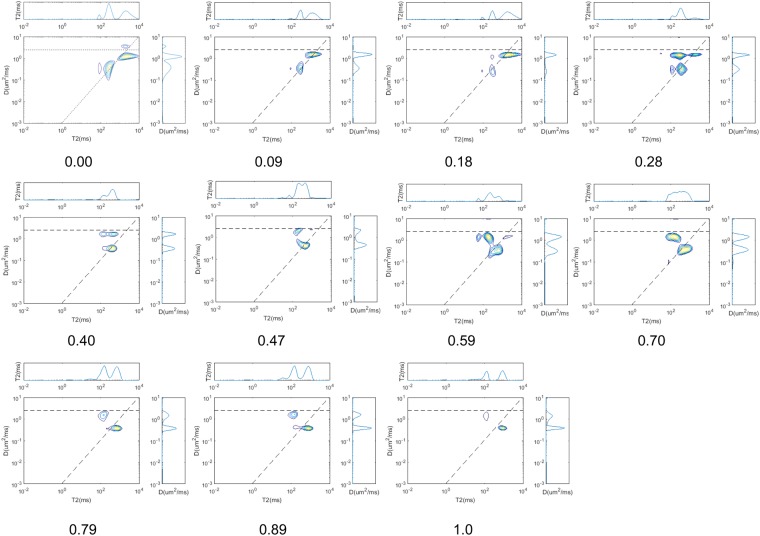
*D* − *T*_2_ maps obtained by inverting the NMR responses simulated using the parameters listed in the last two rows of Table 1 for the fluid distributions in Fig. 5.

**Figure 7:**
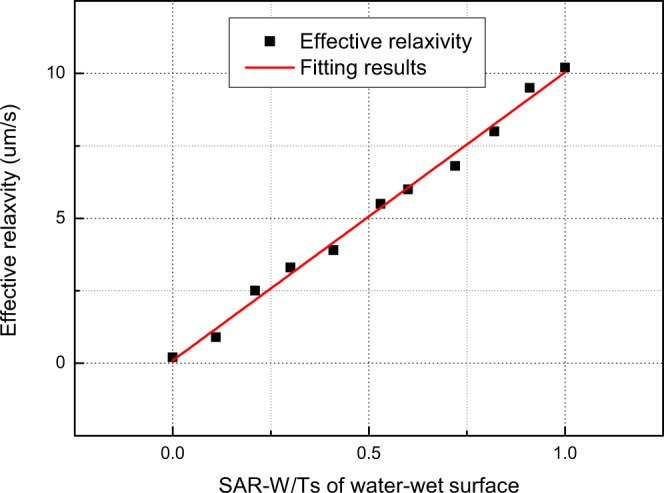
Fitting results between the effective water relaxivities and SAR-W/Ts of water-wet surfaces.

For the NMR response simulation, we employ the random walk method^18^. The method employed to acquire the NMR responses from the obtained oil and water distributions was introduced in section 2.3. The parameters applied during the NMR response simulation are listed in the last two rows of Table 1 ^49^. The amplitude of the pulse field gradient is 0.4 T/m, and its duration is 0.2 ms. The encoding times for diffusion (Δ) are 0.2, 0.8, 1.2, 1.6, 2.0, 2.4, 3.0, 3.6, 4.8, and 6.4 ms. The NMR responses are inverted using a fast Laplace transform^55,56^, the results of which are shown in Fig. 6. The effective relaxivities shown in the *D* − *T*_2_ maps are obtained using the method introduced in section 1.3. The fitting results between the effective water relaxivities and the SAR-W/Ts of water-wet surfaces from the forward models are shown in Fig. 7.

**Figure 8:**
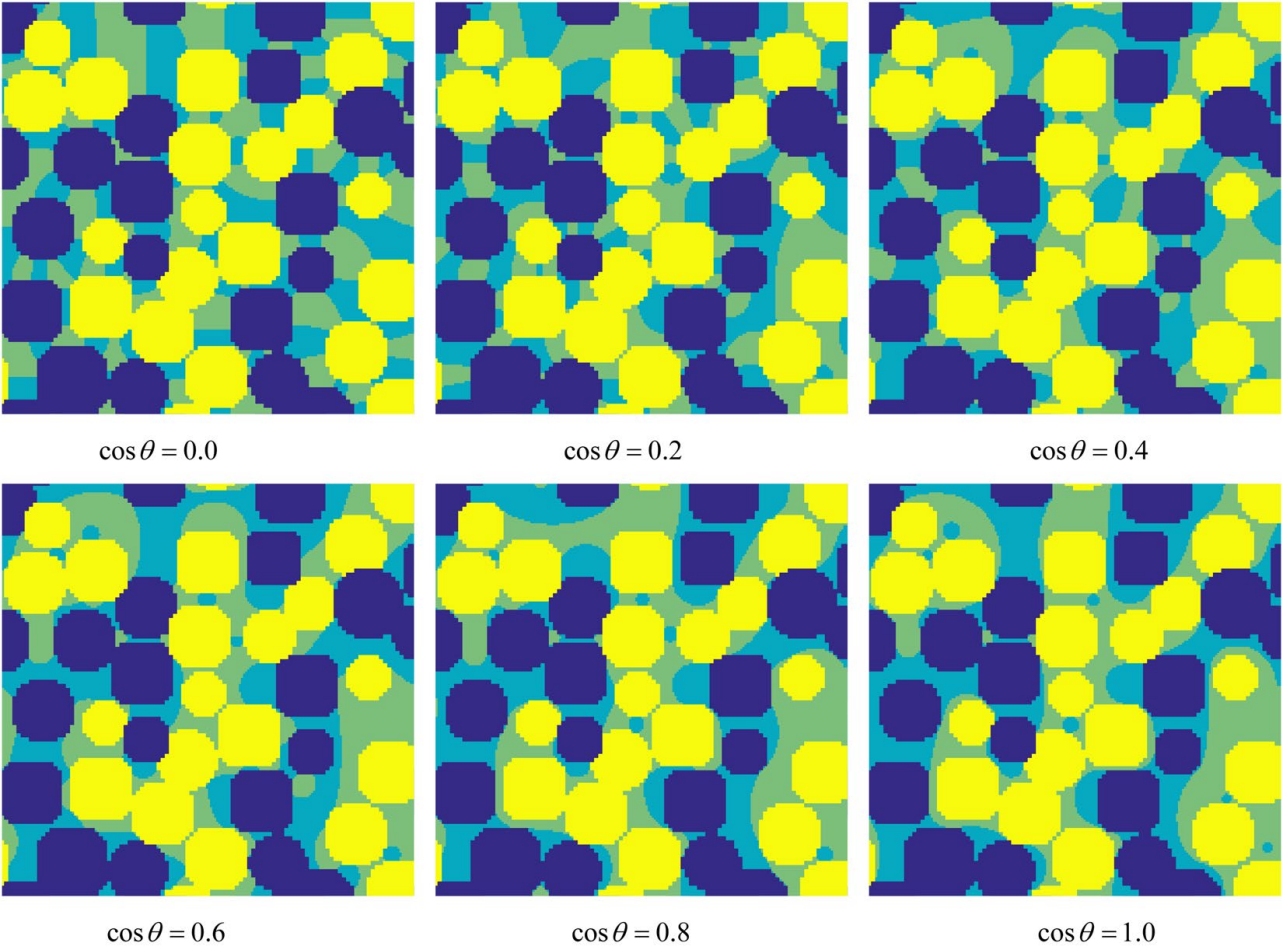
Slices of water and oil distributions in porous media with heterogeneous wettability at *x*/2. The dark blue and bright yellow areas represent water-wet and oil-wet spheres, respectively. The light yellow and light blue parts represent the oil and water distributions, respectively. The SAR-W/T value for the water-wet to oil-wet surface area ratio is 1:1. The cosine values of the contact angles formed by water droplets on the water-wet patches (oil-wet patches) are 0.0 (0.0), 0.2 (−0.2), 0.4 (−0.4), 0.6 (−0.6), 0.8 (−0.8), and 1.0 (−1.0).

For the second part of this section (i.e., investigating the effects of different wetting intensities on the fluid distributions), the SAR-W/T of the water-wet surface is fixed to 0.5. The intrinsic cosine values of the contact angles of the water droplets on the water-wet patches (oil-wet patches) are set to 0.0 (0.0), 0.2 (−0.2), 0.4 (−0.4), 0.6 (−0.6), 0.8 (−0.8), and 1.0 (−1.0). The simulation method and the simulation parameters employed for the fluid distribution simulation are identical to those used for the first part of the simulation except that *G*_*adsw*_ = −*G*_*adso*_. The acquired fluid distributions are shown in Fig. 8.

**Figure 9:**
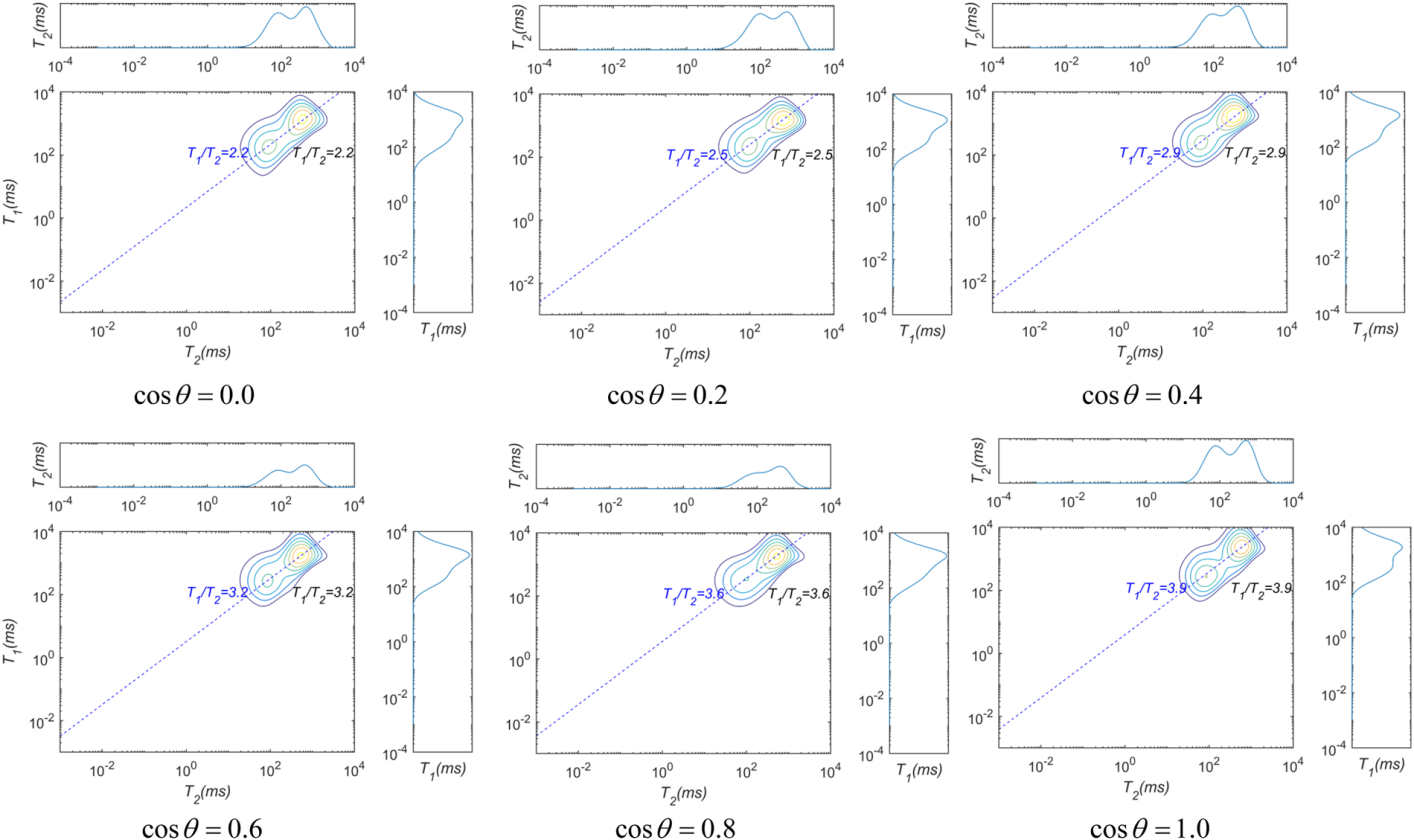
*T*_1_ − *T*_2_ maps for the fluid distributions in Fig. 8. The waiting time follows a logarithmic distribution from 20 ms to 12000 ms with 10 intervals, and the echo spacing is 20 ms. The simulation parameters are listed in the last two rows of Table 1. The *T*_1_ − *T*_2_ values are calculated by linearly fitting the mean *T*_1_ values for fluids on the maps.

**Figure 10:**
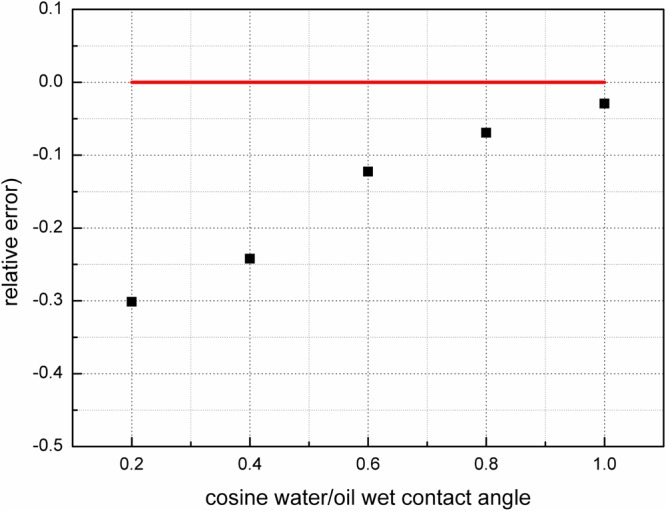
Relative errors between the cosine values of the apparent contact angles using the NMR method and those from the forward modeling. The red line is a theoretical baseline value.

The value of *T*_1_/*T*_2_ is an indicator of the intensity of the interaction between the fluid molecules and the solid surface (i.e., the wettability), as shown in section 1.4. To obtain the *T*_1_ − *T*_2_ maps from *T*_1_ − *T*_2_ − *D*, the NMR responses to the fluid distributions are simulated in porous media using an IR-PFG-CPMG pulse sequence. The waiting time, which follows a logarithmic distribution, ranges from 20 ms to 12000 ms with 10 intervals, and the echo spacing is set to 20 ms. The amplitude of the pulse field gradient is 0.5 T/m, and its duration is 4 ms. The encoding times for diffusion (Δ) are 25 ms. The number of gradients is 10, following a logarithmic distribution. The simulation parameters are listed in the last two rows of Table 1. The *T*_1_ − *T*_2_ maps are obtained by inverting the NMR signals using the fast Laplace transform, and the *T*_1_ − *T*_2_ values are obtained by fitting the mean *T*_1_ values and *T*_2_ for fluids on the *T*_1_ − *T*_2_ map. The inversion results are shown in Fig. 9. The effective relaxivities are obtained from the *D* − *T*_2_ maps. SAR-W/Ts are acquired for an initial value of the surface relaxivity, and the apparent contact angles are computed using equation 2. The errors in the theoretical apparent angles calculated using equation 2 with the proposed NMR measurement method are shown in Fig. 10.

## Discussion

The results in Figs 3 and 4 reveal that the cosine values of the apparent contact angles on flat planes characterized by heterogeneous wettability are linearly proportional to the SAR-W/Ts of the wetting patches and the cosine values of the intrinsic contact angles of each wetting patch. The slope in Fig. 3(b) is 1.89, while that in Fig. 4(b) is 1.14, both of which matching equation 2. The fitting slope between the cosine values of the apparent contact angles and the cosine values of the intrinsic contact angles of the wetting planes from the top two rows of Table 1 in *Huang et al*.^48^ is 1.10, which also matches our result of 1.14. These results validate both the LBM for simulating fluid distributions under the influence of heterogeneous wettability and equation 2. Thus, the macroscopic concept of apparent contact angles is applicable for evaluating the wettability of flat planes with patterned wetting patches.

We apply the concept of apparent contact angles in porous media characterized by heterogeneous wettability to the proposed NMR method. To validate this concept for evaluating heterogeneous wettability using the multidimensional NMR method, a numerical simulation is first performed. First, the fluid distributions are simulated using the SC LBM in porous media with heterogeneous wettability. The results in Figs 5 and 8 prove that the wettability is an important parameter for multiphase fluid distributions in porous media and that the wetting fluid almost comes into contact with the wetting surface. However, with a decrease in the local wettability, the wetting fluid will begin to deviate from the wetting patch, as shown in Fig. 8. This means that the SAR-W/T values obtained using the NMR effective relaxivity deviate from the true SAR-W/Ts under intermediate wetting conditions.

The results from the NMR simulation and inversion are shown in Figs 7 and 10, respectively. Figure 7 shows that *T*2 − *D* can be feasibly applied to obtain the SAR-W/Ts of the wetting patches. Assuming that the cosine values of the wetting contact angles are linearly proportional to the USBM* index, the values of *T*_1_/*T*_2_ are applied to represent the wetting intensities of porous media. Figure 10 demonstrates that the multidimensional NMR approach is applicable for calculating the apparent contact angles in porous media characterized by heterogeneous wettability. However, the relative errors deviate from the baseline primarily when the intrinsic wetting angles of the wetting patches are 0 rad; this is because the wetting patches are characterized by intermediate wetting, i.e., they cannot retain a wetting fluid, as suggested in Fig. 8. The fluid distributions therefore also affect the apparent diffusion coefficients of fluids, thereby contributing to the observed deviation.

These results also confirm that the proposed multidimensional NMR probing method can provide a unique perspective on the characterization of heterogeneous wettability that is different from those obtained using the Amott index or USBM index, both of which only providing an average value. The proposed NMR method can supply the SAR-W/Ts and wetting intensities of wetting patches within porous media. The results reported herein also indicate that methods employed to treat only the effective relaxivity as representative of the wetting index^17,19,21,23^ are not appropriate; this is true because wetting patches in porous media are not entirely water-wet or oil-wet, and thus, these methods neglect the factor of the local wettability of wetting patches.

However, when the wetting intensity of each patch is intermediate, the area ratio of fluids that are in contact with the solid surface is not equal to that obtained using the effective relaxivity method. Thus, caution should be taken when employing this method. We will therefore illustrate the problem of intermediate-wettability patches during the investigation of porous media with heterogeneous wettability in a future paper.
